# Association between Interleukin-1 Gene Single Nucleotide Polymorphisms and Ischemic Stroke Classified by TOAST Criteria in the Han Population of Northern China

**DOI:** 10.1155/2013/961039

**Published:** 2013-08-26

**Authors:** Zheng Zhang, Li-Jun Liu, Chen Zhang, Yong-Peng Yu

**Affiliations:** ^1^Department of Geriatric Internal Medicine, The Affiliated Hospital of the Medical College of Qingdao University, Shandong 266003, China; ^2^Department of Neurology, The Affiliated Hospital of the Medical College of Qingdao University, Shandong 266003, China; ^3^Department of Neurology, Wendeng Center Hospital of Weihai, The Affiliated Hospital of Weifang Medical College, Shandong 266003, China

## Abstract

Increasing evidence suggests that IL-1**β** (C-511T) and IL-1**α** (C-889T) genes polymorphisms are associated with the susceptibility to cardiocerebral vascular disease. In this paper, we investigated the relationships between these polymorphisms and the risk of ischemic stroke (IS) classified by TOAST criteria in the north Chinese Han population. 440 cases of IS and 486 age- and gender-matched controls of Chinese Han population were enrolled. Association study showed that the TT genotype and T allele of IL-1**α**-889 C/T were significantly associated with IS of a large artery atherosclerosis (LAA) (TT: OR = 2.01, 95% CI = 1.34–3.0, and *P* < 0.001; T: OR = 1.44, 95% CI = 1.18–1.78, and *P* = 0.001). However, there was no significant difference in the distribution of IL-1**α**-889 C/T genotypes and allele frequencies between the two subgroups (small-artery occlusion (SVD) and cardioembolism (CE)) of IS and control groups. No significant association was also found between the IL-1**β**-511 TT genotype and T allele (TT: OR = 0.79, 95% CI = 0.56–1.11, and *P* = 0.175; T: OR = 0.83, 95% CI = 0.68–1.01, and *P* = 0.066) and IS as well as subgroups of CE and SVD. Our results implicated that IL-1**α**-889 C/T gene polymorphism might be associated with the susceptibility to IS, especially to IS with LAA, in a north Chinese Han population.

## 1. Introduction

Ischemic stroke (IS) is a devastating and complex clinical syndrome, involving a large array of biological processes and heterogeneous etiologies, which together contribute to the susceptibility to develop and maintain ischemic events. Increasing evidence supports that the main pathogenesis is inflammation [1, 2] and atherosclerosis. It is now well accepted that atherosclerosis is not only a lipid disorder but also a chronic inflammatory syndrome [3]. The role of IL-1 in atherogenesis has been investigated by virtue of influencing its level or activity [4]. The previous study further strengthened that IL-1 signaling played an important role in atherosclerosis [5]. The IL-1 gene family exists in two forms, namely, IL-1*α* and IL-1*β*, and one antagonistic cytokine, the IL-1 receptor antagonist (IL-1Ra) [6]. Both IL-1*α* and IL-1*β*, which are produced by lymphocytes or monocytes in the loci of inflammation, exert similar but not completely overlapping biological functions mediated through the IL-1Ra. IL-1*α* and IL-1*β* contribute to the development of vascular damage and atherosclerosis by stimulating cell proliferation and differentiation and the release of matrix-degrading enzymes.

Human genetic association studies have suggested a potential relationship between variants of the IL-1 gene and IS [7]. Because IS is a heterogenous disease with different etiologic subtypes, it is possible that subtype specificity may contribute to these inconsistent results. Replications of these findings have been conducted in various populations [2, 3, 8–19], and the results are not entirely consistent. The associations between IL-1*β* (C-511T) and IL-1*α* (C-889T) and risk of IS have been studied in previous studies [17], as well as in several studies for the Chinese [20–22]. However, data about the relationship between the IL-1*α* (C-889T) polymorphisms and the risk of IS classified by TOAST criteria (especially for small-artery occlusion (SVD) and cardioembolism (CE)) is limited in China. Therefore, we proceed here to investigate whether there is an association between genetic variation in the IL-1*β* (C-511T) and IL-1*α* (C-889T) and overall IS and/or any etiologic subtypes of IS classified by TOAST in a north Chinese Han population.

## 2. Materials and Methods 

### 2.1. Subjects

The ischemic stroke (IS) group and healthy controls from the same geographic area of Northern China were investigated. All procedures conformed to the tenets of the Declaration of Helsinki. Informed consent was obtained from each subject, and the study was approved by the Institutional Review Board of local region. The IS group consisted of 440 ischemic stroke patients (291 men and 149 women, mean age 66.6 ± 8.4 years) diagnosed by computerized tomography scan and/or nuclear magnetic resonance imaging analysis, who were admitted consecutively into the Department of Neurology, the Affiliated Hospital of Qingdao Medical College between October 2009 and May 2011. The controls consisted of 486 healthy volunteers (314 men and 172 women, mean age 66.1 ± 5.2 years) who came from medical center of hospital. They had no evidence of cardiocerebral vascular disease, autoimmune disease, or tumor. The various laboratory tests as well as transcranial Doppler sonography (TCD), cervical vascular Doppler ultrasonography, computer tomography angiography (CTA), or magnetic resonance angiography (MRA) vascular screening were performed when the patients were admitted to the hospital. Considering that cardioembolism might have a different etiology origin, these patients were excluded from this study. Patients with clinical evidence of autoimmune disease or tumor were also excluded. According to TOAST criteria, IS patients were divided into large-artery atherosclerosis (LAA), small-artery occlusion (SVD), and cardioembolism (CE). The rest of the types were not studied in this experiment [11]. No difference was found in sex distribution between the two groups (*P* = 0.982). There were no relationships between these two groups. Both of them are of Chinese ancestry and come from the northern regions of China.

### 2.2. Genetic Analyses

Genomic DNA was extracted via blood sample from each subject. Genetic variants in the promoter region of the IL-1*β* and IL-1*α* genes were identified by using polymerase chain reaction (PCR) and DNA sequencing in all cases and controls. Based on previous studies [15, 21], two primers were designed and listed in (Table 2), which were used to amplify the polymorphic region by PCR (Shanghai Shenggong Biological Technology Co., Ltd). The PCR products were analyzed on a 1.5% agarose gel stained with ethidium bromide. Gels were visualized under ultraviolet light.

### 2.3. Statistical Analysis

The software SPSS for Windows, version 11.5, was used for statistical analysis. The genotype distributions of all the investigated polymorphisms were test deviations from the Hardy-Weinberg equilibrium. Allele, genotype frequencies were compared between groups using *χ* ^2^ or Fisher exact test. The distributions of gender and smoking were analyzed by using the Mann-Whitney *U* test. Differences in age and blood lipid or glucose levels were tested by using the *t*-test (two groups). Type I error rate of 5% was chosen for the analyses. The ORs and 95% CIs for the effect on IS were determined from multiple logistic regression analysis using gender, age, hypertension, diabetes mellitus, hyperlipidemia, and smoking. *P* < 0.05 was considered statistically significant.

## 3. Results

The main characteristics of the IS patients and controls are listed in (Table 1). The IS patients had a higher proportion of smokers, body mass index (BMI), hypertension, diabetics, and more unfavorable profile of plasma low density lipoprotein cholesterin (LDL). The mean age of cases with LAA was significantly older than that of cases with CE. There was no significant difference in gender among stroke patients with different TOAST subtypes (*P* = 0.22).

The genotype distributions and allele frequencies of the IL-1*α*-889 and IL-1*β*-511 polymorphisms in both groups are shown in (Table 3). There are significant differences in the frequencies of genotypes and T allele of IL-1*α*-889 between the IS group with healthy controls (genotype: *P* = 0.002; T: OR = 1.304, 95% CI = 1.08–1.58, and *P* = 0.006). In terms of stroke etiology, TT genotype and T allele frequencies of IL-1*α*-889 were more common among cases with LAA (cases with LAA versus controls: TT: 18.4% versus 10.1%, *P* < 0.05; T: OR = 1.442, 95% CI = 1.18–1.78, and *P* = 0.001). No significant difference in the genotype and allele frequencies of IL-1*α*-889 was found between cases with SVD and controls (*P* > 0.05). No significant differences were found in the IL-1*β*-511 genotype and allele distribution between case and control groups and among stroke patients with different TOAST subtypes (LAA, CE, and SVD) classified by stroke etiology (*P* > 0.05). 

## 4. Association Analysis

The association study between genetic variants in the promoter region of the IL-1*α*-889 gene and IS is shown in Table 3. The T allele of IL-1*α*-889 was significantly associated with IS. Subgroup analysis indicated that this association was limited to cases with LAA (OR = 1.442, 95% CI = 1.18–1.78, and *P* = 0.001), but not for cases with CE (OR = 0.643, 95% CI= 0.36–1.13, and *P* = 0.121) SVD (OR = 0.797, 95% CI = 0.56–1.13, and *P* = 0.204). No significant association was found between the IL-1*β*-511 genotype and IS (*P* > 0.05).

Logistic regression analysis showed that the TT genotype of IL-1*α*-889 was significantly associated with IS (TT: OR = 2.256, 95% CI = 1.178–4.320, and *P* = 0.014). In terms of stroke etiology, this relationship was especially limited to the LAA patients (adjusted OR = 2.029, 95% CI = 1.067–3.858, and *P* = 0.031), while there was no correlation between IL-1*β*-511 genotype and clinical performance for the three models of inheritance. Adjustment for age, sex, smoking habit, diabetes mellitus, blood pressure, BMI, and blood lipid levels did not change these results.

## 5. Discussion

We report a relative large sample case-control study investigating IL-1*α* (C-889T) and IL-1*β* (C-511T) in overall IS and IS subtypes, and our study suggests an association between genetic variations in IL-1*α* (C-889T) and IS with LAA.

Most of the genes coding for the IL-1 family of proteins and clustered on the 2q12-q21 locus (IL-1*α*, IL-1*β*, and IL-1Ra) are polymorphic in multiple loci [23]. A single nucleotide polymorphism (SNP) of the IL-1*α* gene was located at position −889 in the 59-flanking region, and the other was found at position +4845. Four loci of the gene encoding the IL-1*β* gene polymorphism are −511 locus and −31 locus in the promoter region and +3953 and 5810 loci in the region within exon 5 and intron 4 [24, 25]. The IL-1*α* and IL-1*β* levels in brain tissue were significantly increased in the animal models of IS [26, 27]. When recombinant IL-1*β* was injected into the lateral ventricles or directly into the brain parenchyma in the animal models, the levels of IL-1*α* and IL-1*β* significantly increased, which would result in ischemia or other causes of brain damage [28–30]. These findings suggest that IL-1*α*, IL-1*β* can be involved in the pathophysiology of atherosclerosis process and also participate in brain injury after the IS.

Because IS is a heterogenous disease with different etiologic subtypes, the potential pathogenesis may vary from different types of IS. However, IS patients in Chinese population were not classified by TOAST criteria in the previous study. Based on this fact, we investigate whether there is a relationship between genetic variation in the IL-1*β* (C-511T) and IL-1*α* (C-889T) and other etiologic subtypes of IS classified by TOAST in a north Chinese Han population. Then, this study also added the information about the IS subtype of CE, SVD, and this study adds novel information about the association between SNPs of IL-1 (IL-1*α*, IL-1*β*) and IS with different etiologic subtypes in Chinese Han population. 

In the present study, we found that the IL-1*α*-889 C/T was associated with overall IS in the Han Chinese population. The main finding was that the association between the TT genotype and T allele of IL-1*α*-889 and IS was mainly limited to stroke patients with LAA, but not for those cases with CE, SVD. The present data also indicated that IL-1*α*-889 TT carriers were associated with a significantly increased risk of IS compared with patients without IL-1*α*-889 TT carriers. However, we did not find a difference in the IL-1*β* (C-511T) polymorphism between cases and controls. These results were consistent with previous studies [3, 11, 12, 14, 15]. A previous study indicated that the IL-1*α*-889 TT genotype significantly increased the transcriptional activity of the IL-1*α* gene with respect to the CC genotype. A slight increase of the IL-1*α* mRNA and protein levels was also detected in the plasma [31]. An SNP in the promoter region of IL-1*β* at position −511 resulting in C-T transition influenced the protein production, and IL-1*β*-511T carriers were reported to be higher producers of IL-1*β* than IL-1*β*-511C carriers [12]. While based on these preliminary results, we could not provide evidence for causal molecular mechanisms of the TT genotype of IL-1*α*-889 for increased risk of IS, we cannot exclude the possibility that IL-1*β* polymorphism (C-511T) could interact with other cytokines, which always work in a network. Therefore, a genetic predisposition to produce anti-inflammatory cytokines (e.g., IL-10 or IL-1Ra) could interfere with the biological effects of IL-1. With regard to the selected participants for comparing serum IL-1*α* and IL-1*β* concentrations, we could not determine whether there was a high proportion of stroke patients with CE, SVD, or any other determined etiology. This might enlighten us to investigate the relationship between variants of the IL-1 gene cluster, the serum concentration of IL-1 and IS according to stroke etiology in the future. 

There were some limitations in this study. First, this study was not a big enough sample study. The power size is limited for subgroups analysis according to stroke etiology. Second, the cases that either died or were too ill were not enrolled in this study. It is difficult to predict what effect this selection bias may have. Further genetic studies on patients who did not survive after the ischemic event may answer the question whether IL-1*α*-889 C/T individuals are also exposed to greater mortality. Other limitations of this study are that we did not investigate blood levels or monocyte production of IL-1*α* or IL-1*β* as well as the mechanisms of action such as altered IL-1*α* or IL-1*β* gene expression or alternative splicing by the IL-1*α*-889 C/T or IL-1*β*-511 C/T SNPs. Thus, further investigation regarding the genetic effect on IL-1*α* and IL-1*β* mRNA structure and mRNA expression levels and cytokines levels would strengthen the results of this study. This might have provided some mechanistic insights into the relationship between variants of the IL-1*α* and IL-1*β* genes and IS according to stroke etiology. A large number of basic and clinical researches on the polymorphisms of IL-1*α*-889 C/T and IL-1*β*-511 C/T had been performed, while the conclusions were not entirely consistent with each other, which may be related to racial differences, research methods, and sample size. The present study also lacked data on drinking habits, and the IS subtype of cryptogenic stroke was not included in the present study. Last but not least, we cannot confirm whether IL-1*α*-889 TT can interact with or be in linkage disequilibrium with other functionally important genes, which could be involved with the susceptibility of IS. These questions need confirmation in other studies.

## 6. Conclusion 

The results reported here suggest a significant association between IL-1*α*-889 C/T polymorphisms and the susceptibility to IS, especially to IS with LAA, in a north Chinese Han population. However, in our study, we found no relation between IL-1*β*-511 C/T polymorphism and LAA, SVD, or CE. Our results require replication/confirmation in larger examples and more strictly designed case-control studies in the future.

## Figures and Tables

**Table 1:** Demographics, risk factors, and stroke etiologies of IS and control groups.

	IS (*n* = 440)	Control (*n* = 486)	*P*
Demographics			
Age (±SD)	66.6 ± 8.4	66.1 ± 5.2	0.287
Male (%)	291 (66.1)	314 (64.7)	0.626
Risk factors			
Smoking (%)	121 (27.5)	98 (20.2)	0.009
Body mass index (BMI) (Kg·m^−2^) (mean ± SD)	24.9 ± 3.6	24.8 ± 3.1	0.002
Hypertension (%)	262 (59.5)	244 (50.2)	0.004
Diabetes mellitus (DM) (%)	126 (28.6)	62 (12.8)	<0.05
LDL (mmol/L) (mean ± SD)	3.0 ± 0.8	3.0 ± 0.6	0.03
Large arterial atherosclerosis (LAA), *n* (%)	320 (72.7)		
Cardioembolism (CE), *n* (%)	32 (7.3)		
Small vessel disease (SVD), *n* (%)	88 (20.0)		

**Table 2:** The main characteristics of IL-1 gene and techniques used for screening.

	IL-1*α*	IL-1*β*
Polymorphism type	Single base C/T	Single base C/T
Polymorphism site	−889	−511
PCR primers		
Upstream	5-GGGGGCTTCACTATGTTGCCCA CACTGGACTAA-3	5-TGGCATTGATCTGGTTCATC-3
Downstream	5-GAAGGCATGGATTTTTACATAT GAGCCTTCCATG-3	5-GTTTAGGAATCTTCCCACTT-3
PCR condition		
Denaturation	94°C	94°C
Annealing	57°C	55°C
Extension	72°C	72°C
Cycles (*n*)	35	35
Digestion	NCOI	AvaI
Fragment size (pb)	295	304

**Table 3:** Genotype and allele frequencies of variants in the IL-1*α*-889 and IL-1*β*-511 gene, and association with ischemic stroke.

Groups	IL-1*α*-889 genotype					IL-1*α*-889 allele			
	TT	CT	CC	*P*	TT versus CT + CC *P*, OR, 95% CI	*C*	*T*	*P*	OR, 95% CI
Control, *n* = 486	49 (10.1)	237 (48.8)	200 (41.1)			637	358		
IS, *n* = 440	63 (14.3)	232 (52.7)	145 (33.0)	0.016	0.065 1.45, (0.97–2.15)	522	335	0.006	1.30, (1.08–1.58)
LAA, *n* = 320	59 (18.4)	158 (49.4)	103 (32.2)	0.001	<0.05 2.01, (1.34–3.0)	364	276	0.001	1.44, (1.18–1.78)
SVD, *n* = 88	4 (4.5)	44 (50.0)	40 (45.5)	0.243	0.098 0.42, (0.15–1.17)	124	52	0.204	0.80, (0.56–1.13)
CE, *n* = 32	0 (0)	16 (50.0)	16 (50.0)	0.15	0.115 (—)	48	16	0.121	0.63, (0.36–1.13)

	IL-1*β*-511 genotype					IL-1*β*-511 allele			
	TT	CT	CC	*P*	TT versus CT + CC *P*, OR, 95% CI	*C*	*T*	*P*	OR, 95% CI

Control, *n* = 486	117 (24.1)	261 (53.7)	108 (22.2)			477	495		
IS, *n* = 440	95 (21.6)	226 (51.3)	119 (27.1)	0.217	0.369 0.87, (0.63–1.18)	464	416	0.116	0.86, (0.72–1.04)
LAA, *n* = 320	64 (20.0)	168 (52.5)	88 (27.5)	0.158	0.175 0.79, (0.56–1.11)	344	296	0.066	0.83, (0.68–1.01)
SVD, *n* = 88	20 (22.7)	43 (48.9)	25 (28.4)	0.446	0.785 0.93, (0.54–1.6)	93	83	0.358	0.86, (0.62–1.19)
CE, *n* = 32	6 (18.8)	15 (46.9)	11 (34.3)	0.424	0.392 0.73, (0.29–1.8)	27	37	0.286	0.29, (0.80–2.20)
